# Influence of different photoinitiators on the resistance of union in bovine dentin: Experimental and microscopic study

**DOI:** 10.4317/jced.57756

**Published:** 2021-02-01

**Authors:** Marcelo Basílio, Renata Gregorio, João-Victor Câmara, Lizandra Serrano, Paulo-Ricardo Campos, Josué-Junior Pierote, Sonia Groisman, Gisele Pereira, Suelem Barreto

**Affiliations:** 1 MSc, Department of Dental Clinic, School of Dentistry, Federal University of Rio de Janeiro, Rio de Janeiro, RJ, Brazil; 2 DDS, Department of Dental Clinic, School of Dentistry, Federal University of Rio de Janeiro, Rio de Janeiro, RJ, Brazil; 3 Master student, Department of Biological Sciences, Bauru School of Dentistry, University of São Paulo, Bauru, São Paulo, Brazil; 4 DDS, Department of Dental Clinic, School of Dentistry, Federal University of Rio de Janeiro, Rio de Janeiro, RJ, Brazil; 5 Professor, Department of Dentistry, University of Santo Amaro, São Paulo, SP, Brazil; 6 Full Professor, Department of Social and Preventive Dentistry, Federal University of Rio de Janeiro, Rio de Janeiro, RJ, Brazil; 7 Adjunct Professor, Department of Dental Clinic, School of Dentistry, Federal University of Rio de Janeiro, Rio de Janeiro, RJ, Brazil; 8 Professor, Department of Dental Clinic, School of Dentistry, Federal University of Rio de Janeiro, Rio de Janeiro, RJ, Brazil

## Abstract

**Background:**

To evaluate *in vitro* the influence of photoinitiators on the microtensile strength of adhesive systems and composite resins in bovine dentin.

**Material and Methods:**

Forty dentin obtained from bovine teeth were randomly distributed in four groups (n = 10) according to the different adhesive systems and composite resins used: G1 - AAPS + VAPS (Ambar APS + Vittra APS); G2- AAPS + O (Ambar APS + Opallis); G3 - A + VAPS (Ambar + Vittra APS) and G4 - A + O (Ambar + Opallis). After restoration with the composite, the samples were sectioned to obtain toothpicks that were subjected to the microtensile and nanofiltration test (1.0 mm/min).

**Results:**

The Kruskal-Wallis test did not show significant differences between the groups (*p*<0.05). The values in MPa were: AAPS + VAPS - 19.56 MPa; AAPS + O - 19.77 MPa; A + VAPS - 17.78 MPa; A + O - 22.44 MPa. The result of the Mann-Whitney test showed no significant differences depending on the adhesive (Ambar Universal- 19.11 MPa, Ambar APS Universal- 21.70 MPa) and the composite resin used (Vittra APS- 18.75 MPa, Opallis - 23.75 MPa). The AAPS + VAPS and AAPS + O groups showed intense silver nitrate infiltration. The A + APS group showed a moderate infiltration and the A + O group had a mild infiltration in the adhesive system/dentin interface.

**Conclusions:**

The use of different photoinitiators in the composition of adhesive systems and restorative composites did not affect their bond strength values and the presence of water in the solvent of the APS photoinitiator system had a negative influence, increasing the degree of infiltration in the hybrid layer when compared to the camphorquinone photoinitiator.

** Key words:**Dentin, adhesive systems, composite resin, photoinitiators, tensile strength, nano-infiltration.

## Introduction

Adhesive systems have been improved for over 50 years and have developed in such a way that their application in current dentistry has become common and reliable.1 They are used in several adhesive procedures such as the production of direct restorations, cementation of intraradicular posts, and fixation of prosthetic parts (1,2).

The main challenge for dental adhesives is to provide an equally effective bond to two hard dental substrates of different types and still provide retention to resin composites under the forces exerted by the masticatory loading and polymerization shrinkage of these materials. To ensure enhanced properties, adhesive systems are constantly improving, from reducing the number of steps to formulating new constituents (3,4)

Recently, the APS (Advanced Polymerization System) was launched in the market, consisting of a combination of different photoinitiators that interact with each other, amplifying the light activation capacity emitted by the photopolymerization units. Added to different materials, the system offers different advantages and the main benefit is the increase in the degree of conversion in the hybrid layer, which increases bond strength and consequently the mechanical properties of the adhesive film (higher cohesive strength) (1,2). Another advantage is the absence of color in this system, preventing any type of interference when performing restoration/cementation in anterior teeth. Thus, it is observed that simplified adhesive systems, also known as single-bottle adhesives, have been extensively used because of their agility and ease of application, reducing the clinical time for adhesive restorative procedures. Evaluating the properties of these systems is necessary due to their extensive use and, considering the application time, the optimization of the polymerization process might positively affect the mechanical properties of these materials, improving their performance in the clinical steps (5,6).

Thus, this study aimed to evaluate the microtensile strength between adhesive systems and composite resins with different types of photoinitiators, as well as the infiltration in the adhesive system and dentin substrate interface. The null hypothesis was that the APS photoinitiator system does not affect the bond strength of adhesive systems and composite resins based on APS or camphorquinon in bovine dentin.

## Material and Methods

-Ethical aspects

To conduct this research, approval was requested to the Ethics Committee for the Use of Animals in Scientific Experiments, from the Health Sciences Center of the Federal University of Rio de Janeiro (CCS/UFRJ), which was accepted by process n° 010-19.

-Materials

The following restorative materials were used in this study: 37% phosphoric acid FGM, Ambar and Ambar APS adhesive systems, Opallis and Vittra APS composite resins, and the Valo photoactivator.  Table 1 describes the composition and manufacturers of the materials used in the study.

**Table 1:**
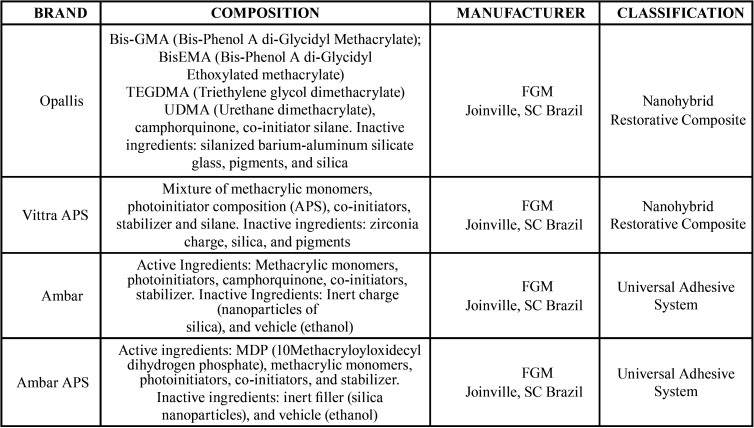
Trademark, components and manufacturers of the materials used in restorative procedures.

-Experimental groups

Forty bovine incisors (n = 10) were randomly divided into experimental groups formed by the interaction between adhesive systems and composite resin: Group 1 - Ambar APS and Vittra APS; Group 2 - Ambar APS and Opallis; Group 3 – Ambar and Vittra APS; and Group 4 – Ambar and Opallis.

-Teeth preparation

Forty healthy bovine incisors were stored for a maximum of one month in 0.1% thymol solution, at room temperature (UFRJ– CCMN- Department of Biochemistry, Rio de Janeiro- RJ- Brazil) and pH 7.0, for disinfection. The external surfaces were cleaned with a Cavitron ultrasound system (Dentsply, RJ, Brazil) and periodontal curettes (Duflex, SSWhite, Rio de Janeiro, RJ, Brazil). After cleaning, the teeth were stored in distilled water until the surfaces were prepared.

Then, the root portion was separated from the coronary portion close to the amelocemental junction (ACJ) by a double-sided diamond cutting disc (Dhpro, Barueri, SP, Brazil) mounted in a straight piece. The palatal surfaces of all teeth were accessed with a diamond tip number 1014L (KG Sorensen Ind. E Com. Ltda, Barueri, SP, Brazil) mounted at high rotation (Kavo, SA Ind. Com. Ltda, Joinville, SC, Brazil) to fill the pulp chamber with composite resin to increase the thickness of the tooth in the thinner region of the pulp chamber.

After preparing the palatal surface, the buccal surfaces of the samples were flattened in a water-cooled rotating electric polisher (Aropol VV, Arotec, Cotia, SP, Brazil) sequentially with silicon carbide (SiC) sandpapers of #320 and #400 granulations (3M, SP, Brazil) until dentin exposure. To standardize the smear layer, the SiC sandpaper of #600 granulation was used for 15 seconds and the crowns were stored in distilled water in an oven at a temperature of 37ºC until starting the restorations.

-Restorative procedure

The dentin was etched with 37% phosphoric acid (Condac 37%, FGM - Joinville, Brazil) for 30 and 15 seconds, respectively. Then, the surface was washed abundantly with water for 30 seconds and dried by capillarity with the aid of a paper filter (Melitta do Brasil-Ind. E Com. Ltda, Avaré, SP, Brazil). Next, a drop of the adhesive system (Ambar APS or Ambar) was applied according to the experimental group, using a microbrush (Cavibrush, FGM - Joinville, Brazil) for 10 seconds, followed by the application of a new adhesive layer with a microbrush for another 10 seconds, actively and uniformly, in sequence, evaporating it with a mild air spray for 10 seconds, 20 centimeters away.

Subsequently, photoactivation was performed for 10 seconds with the active tip of the device juxtaposed to the surface. The teeth were restored aided by a metallic spatula for composite resin (Suprafill - Duflex / SSWhite - Rio de Janeiro, RJ, Brazil) with resin composite (Vittra APS or Opallis - Color DA2), according to the experimental group, in a 4-mm high, 8-mm long, and 6-mm wide area. Each increment was photoactivated for 10 seconds with a Valo curing device (Valo Cordless, Ultradent - Salt Lake City, Utah, USA) in standard mode - 1,000 mW/cm2, which was measured on a radiometer before each photoactivation. The samples were then immersed in distilled water without the addition of antimicrobials, in which they remained for 24 h for the microtensile test.

-Obtaining specimens for the microtensile test and nano-infiltration analysis

The dental crowns were fixed individually by the palatal portion in acrylic plates, using utility wax (Horus, Herpo Produtos Dentários, Petrópolis, RJ, Brazil) to position the buccal surface of the samples worn in parallel with the acrylic plate. Later, sticky wax (NewWax, Thechnew Com. and Ind. Ltda., Rio de Janeiro, RJ, Brazil) was used for better fixation. The set was properly fixed and adapted to a precision metallographic cutter (IsoMet, Buehler Ltda. Lake Bluff, IL, USA), in which a metal disc (Extec Corp., Enfield, CT, USA) rotating at low speed (300 rpm) and under constant irrigation with distilled water performed serial cuts in the mesiodistal direction to obtain slices with a thickness of 1.0 mm. After repositioning the tooth, cuts were made in the buccolingual direction to obtain toothpicks of approximately 1.0 x 1.0 mm.

-Mechanical test for microtensile strength and nano-infiltration

The dimensions of the adhesive interface for the specimens obtained from serial cuts were measured with the help of a digital caliper (Utustools professional MT-00855, USA) to calculate the area. The specimens were fixed by their ends to position them parallel to Geraldeli’s device (Odeme Biotechnology, Joaçaba, SC, Brazil), aided by a cyanoacrylate adhesive glue (Super Bonder– Henkel Loctite adhesives Ltda, Itapevi, SP, Brazil) for the microtensile test. The apparatus was coupled to the Universal Testing Machine (Instron 33R 5567; Instron Corp., Grove City, PA, USA) and the test was conducted with a 20-kN load cell at a speed of 1.0 mm/min until rupture. At the time of fracture, the movement was immediately stopped. The load required for the fracture of each specimen, in kilogram-force (kgf), was noted and the fracture strength in MegaPascal (MPa) was calculated according to the mathematical formula: R = F (kgf) x 9.8/A (R = bond strength in MPa, F = force in kilogram-force (kgf), and A = area in mm2).

-Production of specimens for the nano-infiltration analysis

Ten toothpicks from each group were randomly selected, immersed in an ammoniacal silver nitrate solution for 24 hours, stored in an oven at 37°C, and protected from the light. After 24 hours, each toothpick was washed under running water and then placed in a developing solution (Kodak Professional D76 Developer, Eastman Kodak Company, USA) under fluorescent light for 8 hours. Afterward, the toothpicks were washed in distilled water. The toothpicks were then covered with polystyrene resin and polymerized for 5 hours.

After the inlay, they were polished in a #600 sandpaper for 2 minutes, a #1200 sandpaper for 10 minutes, and placed in an ultrasonic bath for 5 minutes. Then, the toothpicks were polished with #2000 sandpaper for 10 minutes, placed in an ultrasonic bath for 5 minutes, and polished with TOP felt and alumina paste (0.6 Nm = 6) for 15 minutes. Next, they were placed in an ultrasonic bath for 10 minutes and polished with RAM felt and alumina paste (0.3 Nm = 3) for 15 minutes; then placed in an ultrasonic bath for 10 minutes, polished with SUPRA felt and alumina paste (0.05 Nm = ¼) for 15 minutes, and placed in an ultrasonic bath for 10 minutes. The toothpicks were cleaned with 85% phosphoric acid for 10 seconds and washed with distilled water for the same time. Then, washed with 2% sodium hypochlorite for 10 minutes and placed for 10 minutes in an ultrasonic bath. Dehydration was performed in ascending ethanol concentrations (25% for 10 minutes, 50% for 10 minutes, 75% for 10 minutes, 90% for 10 minutes, and 100% for 10 minutes) and the toothpicks were stored in a recipient with silica gel.

-Scanning Electron Microscopy analysis

After performing the infiltration procedure of the tracer solution, the specimens were coated with a thin layer of gold and examined in a Scanning Electron Microscope (SEM) (Versa 3D Dual Bean), at a power of 20 kV and a focal distance of 9 mm. Secondary electron images were obtained at 650x and 1500x magnifications.

-Statistical analysis

After the Shapiro-Wilks test to assess normality, a deviation from normality was observed. Due to this deviation, non-parametric tests were used to assess the differences between groups. When the analysis involved more than two groups, the Kruskal-Wallis test was used. As there were no significant differences between the groups, no subsequent two-by-two analysis was performed (post-hoc). When comparing two sets of data (different resins and adhesives), the Mann-Whitney test was used. All statistical analyses were performed using the R Project 3.5 software (R Foundation for Statistical Computing, Vienna, Austria) and the level of significance was *p*≤0.05.

## Results

**Figure 1:**
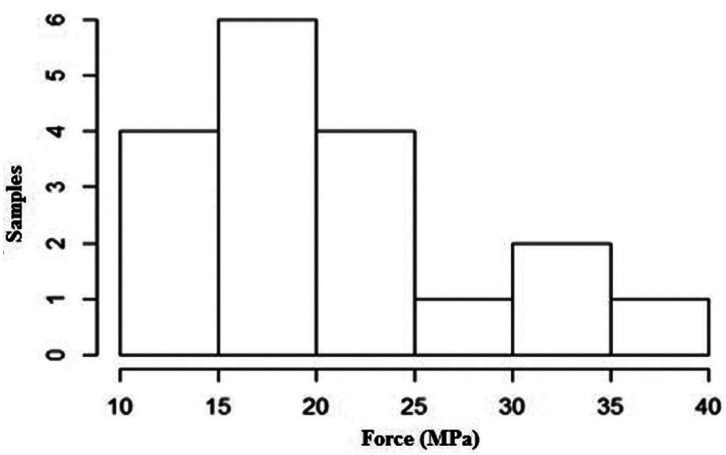
Histogram showing a deviation from data normality.

The data from the Shapiro-Wilks test showed a deviation from normality (Fig. 1). The Mann-Whitney test showed that the “adhesive” (ρ = 0.86) and “composite resin” (ρ = 0.15) factors did not show a statistically significant difference when evaluating bond strength. When the interaction between the factors (“adhesive” x “resin”) was evaluated (ρ = 0.49), the Kruskal-Wallis test did not show statistical differences between the groups ( Table 2- Table 4).

**Table 2:**
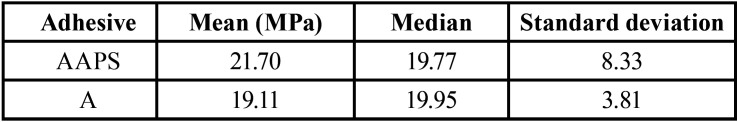
Microtensile strength values (MPa) for the adhesive factor.

**Table 3:**
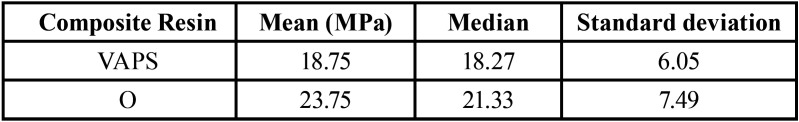
Microtensile strength values (MPa) for the composite resin factor.

**Table 4:**
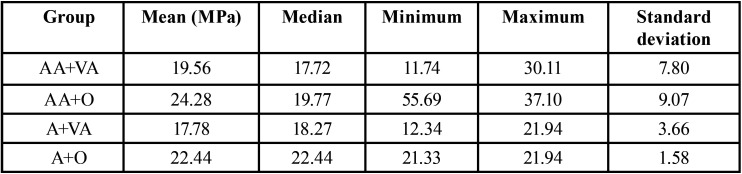
Microtensile strength values (MPa) of the adhesive and resin interaction.

**Figure 2:**
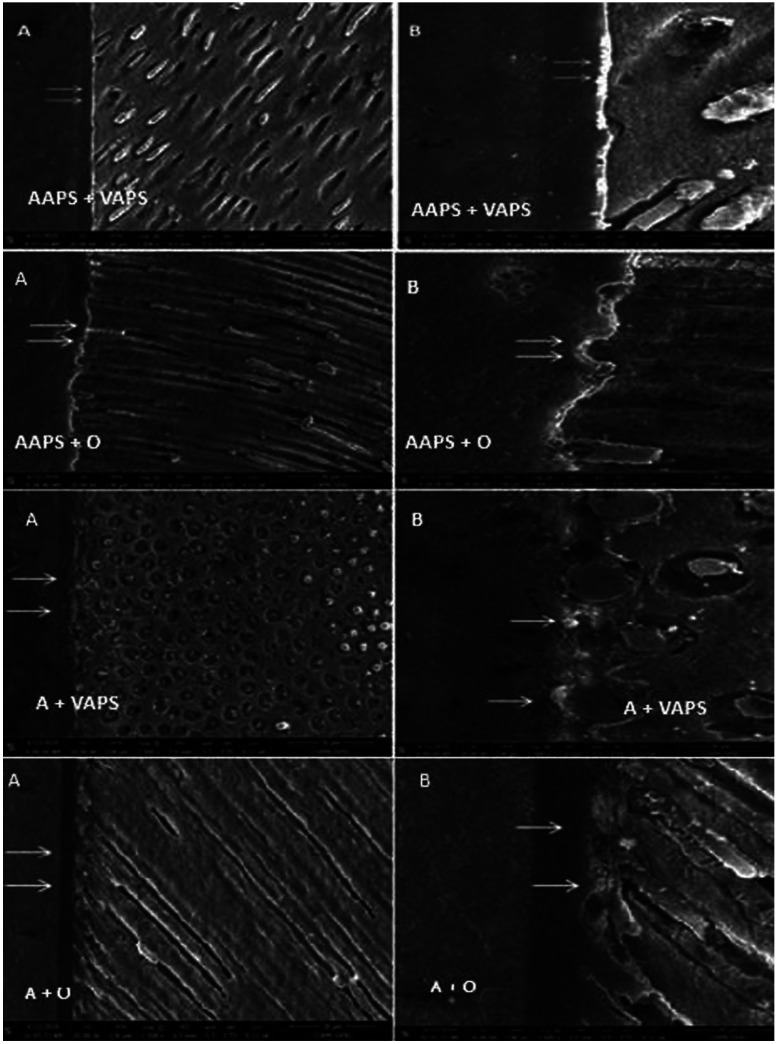
Scanning electron microscopy (SEM) representative of the groups illustrating nanoinfiltration. The white arrows indicate the infiltration of silver nitrate into the gaps in the hybrid layer, where the AAPS + VAPS and AAPS + O group present intense infiltration, the group A + VAPS has moderate infiltration and group A + O has mild infiltration. A) Smallest increase (650X) and B) Highest increase (1500X).

The AAPS + VAPS and AAPS + O groups, both with the APS adhesive system, found an intense silver nitrate infiltration in the hybrid layer. In the A + VAPS group with the Ambar universal adhesive system, moderate infiltration was observed. In the A + O group, also with the Ambar universal adhesive system, a mild infiltration with silver nitrate was observed in the hybrid layer (Fig. 2).

## Discussion

The null hypothesis that the APS photoinitiator system does not affect the bond strength of adhesive systems and composite resins based on the same photoinitiator system or camphorquinone in bovine dentine was accepted. The adhesive systems present satisfactory adhesion to the enamel, however, obtaining a stable bond to the dentin is more difficult and less predictable due to its heterogeneous and physiologically dynamic structure composed of 20% of water (7-9). The establishment of adhesion to the dentin substrate occurs through the replacement of the inorganic material of the dentin substrate by resinous monomers, called hybridization (10).

Hybridization may be affected by the presence of residual water (24) or the lack of solvent evaporation (11) and thus interfere with the mechanical properties of adhesive systems. A quality hybridization process requires an adequate volatilization of water and organic solvents before polymerization (12-14).

The presence of residual solvent interposed to the monomers after photoactivation hinders their interaction and impairs the propagation and growth of polymeric chains (15), providing an incomplete polymerization of resin monomers and creating porosities in the hybrid layer. This favors nano-infiltration (16-18) and results in the loss of mechanical properties after a relatively short clinical period (19,20). Moreover, if solvent evaporation is insufficient, it may dilute the comonomers and interfere with the quality of adhesion (19).

Some clinicians use simplified adhesive systems to shorten the service time. Thus, prior acid etching should be followed by the application of hydrophilic and hydrophobic monomers in the same bottle (21). After this application, the solvent must be correctly volatilized with air (22), however, neglect often occurs in this stage, resulting in an incomplete adhesive infiltration, and thus, some collagen fibrils remain naked (23). Any residual solvent that is not properly volatilized, especially water, interferes negatively with polymerization (8,22,24,25). This failure provides a hybrid layer rich in water and organic solvent (11), leading to lower mechanical properties due to the increased porosity of the polymerized adhesive layer, causing and propagating microfractures, which reflects in the reduction of bond strength values (16,17,26), loss of retention, and marginal mismatch (16).

There was no statistically significant difference between the adhesives ( Table 2) but, numerically, the Ambar APS system showed higher bond strength. This probably occurred due to the application of two layers of adhesives to the dental substrate, as suggested by the manufacturer, indicating the volatilization with air only after the application of the second layer. Thus, the values obtained by the Ambar APS system were not affected, considering it is composed of water and ethanol, preserving dentin moisture. Conversely, the Ambar system presents only ethanol as a solvent, having a higher vapor pressure than water, which allows better evaporation in a shorter period and the collapse of collagen fibrils, making them more rigid and reducing the interfibrillar spaces. This complicates adhesive infiltration and compromises the bonding of adhesive systems to the dentin substrate (8,22,24).

Additionally, the composition of composite resin is closely related to satisfactory physical and mechanical properties. Thus, the type and size of the charged particles, the type of monomer present in the resin matrix, the concentration and/or type of activators, initiators, and inhibitors, and even GC after polymerization may interfere with the quality of the material (27,28). The polymerization depth and GC are closely linked to the ability of light to penetrate through the composite resin, which is determined by its translucency and the presence and type of charge (29). Thus, the characteristics of the inorganic matrix in dental composites have a great impact on the polymeric conversion of these materials (4,24,29). The type, size and concentration of the charge may affect considerably the ability of light to be transmitted through the layer of composite resin (4,24,29). The Vittra APS resin is a nanoparticulate and radiopaque composite with a charge composed of nanospheres of a zirconia complex, with an average particle size of 200 nm and a total charge content of 52 to 60% of volume. As the manufacturer states, the GC of this material is approximately 50 to 60% of the total, which is considered satisfactory. In turn, the Opallis resin is a nano-hybrid composite with barium aluminum silicate glass particles combined with silicon dioxide nanoparticles, ranging from 40 nm to 3.0 μm in size, with an average size of 0.5 μm and a lower load volume than the previous resin; it also presents unparalleled optical properties, according to the manufacturer.

It is suggested that the larger the load size in the composition of composites, the lower its concentration and, consequently, the greater the volume of the resinous matrix, which results in a greater reactive portion, increasing its polymerization and providing greater translucency.5 Additionally, the presence of radiopaque zirconia nano-spheres may have caused a lower monomeric GC of Vittra APS resin due to the higher refractive index, which results in lower numerical retention values than the Opallis resin ( Table 3).

Conversely, standardizing the other factors that affect directly the polymerization of the material was essential for obtaining results in all the groups evaluated. Thus, translucent enamel composites of color A2 were applied to the dentin substrate in 2-mm thick layers, photoactivated with standardized exposure time and with the shortest possible distance from the active tip of the curing light to the material (30). Hence, the lighter color of the resins applied in thin layers favored the deeper penetration of light. Moreover, the high light intensity and the correct level of irradiance were ensured by the exposure time, proximity to the light beam, and type of the light-curing device used (6).

Among these factors, possibly the most important were the wavelength and light intensity provided by the photopolymerizer used in this experiment. The Valo Cordless device (Ultradent - Salt Lake City, Utah, USA) was used for the time indicated by the manufacturers of the materials evaluated, in standard mode. This device uses diodes emitting collimated beams of LED light to produce high-intensity light of approximately 1,000 mW/cm2, with a wavelength ranging between 395 and 480 nm. It is wide-ranging and sufficiently effective for activating the materials used in the present study, greatly affecting the homogeneous pattern of the results obtained. Thus, the need for further studies is evident, changing the variables hereby evaluated, especially concerning the light source used, considering this does not represent the reality found in the public service and most Brazilian dental offices.

The photoactivation reaction is affected by the solvent contained in the adhesive. The ethanol in the Ambar Universal system present in groups A + VAPS and A + O increases the degree of monomeric conversion through the greater mobility of the polymer chain and the greater degree of diffusion of free radicals. However, the water in the Ambar APS Universal system, even when associated with ethanol, interferes in the polymerization process because it dilutes and decreases the reactivity of resin monomers.

Moreover, after its application, the solvent should be correctly volatilized with air (13). Any residual solvent that is not properly volatilized, mainly water, interferes in a negative physical way with polymerization (13,23). Besides the problems previously mentioned, the presence of the solvent increases the porosity of the polymerized adhesive layer, causing and propagating microfractures, which reduces bond strength values. The manufacturer of the two adhesive systems evaluated suggests applying two layers on the dental substrate, indicating the volatilization with air only after applying the second layer. This fact possibly did not affect the interface obtained by the Ambar Universal/dentin system, considering the solvent in its formulation is ethanol, which has a higher vapor pressure than water and allows better evaporation in a shorter time (13). However, it may have interfered with the strength values of the Ambar APS Universal systems due to the presence of water in its composition.

In the case of primers with aqueous solvents, the presence of hydrophilic components such as photoinitiators and monomers may hinder the volatilization of water and promote its excess in the hybrid layer (25).This does not occur with camphorquinone, because it is hydrophobic, that is, it has no affinity with the water in the solvent, thus facilitating evaporation. Therefore, the photoinitiator system should be selected according to the composition of the adhesive system, considering the presence of residual water and/or solvents.

## Conclusions

- The interaction of the APS or camphorquinon photoinitiators in the adhesive system and the restorative composite did not affect microtensile strength values.

- The APS photoinitiator system showed the same behavior on bond strength when compared to camphorquinon.

- The presence of water in the solvent of the APS photoinitiator system had a negative effect, increasing the degree of infiltration in the hybrid layer when compared to the camphorquinone photoinitiator.
